# A luciferin analogue generating near-infrared bioluminescence achieves highly sensitive deep-tissue imaging

**DOI:** 10.1038/ncomms11856

**Published:** 2016-06-14

**Authors:** Takahiro Kuchimaru, Satoshi Iwano, Masahiro Kiyama, Shun Mitsumata, Tetsuya Kadonosono, Haruki Niwa, Shojiro Maki, Shinae Kizaka-Kondoh

**Affiliations:** 1 School of Life Science and Technology, Tokyo Institute of Technology, 4259, Nagatsuta, Midori-ku, Yokohama 226-8501, Japan; 2 Graduate School of Informatics and Engineering, The University of Electro-Communications, 1-5-1 Chofugaoka, Chofu, Tokyo 182-8585, Japan

## Abstract

In preclinical cancer research, bioluminescence imaging with firefly luciferase and D-luciferin has become a standard to monitor biological processes both *in vitro* and *in vivo*. However, the emission maximum (*λ*_max_) of bioluminescence produced by D-luciferin is 562 nm where light is not highly penetrable in biological tissues. This emphasizes a need for developing a red-shifted bioluminescence imaging system to improve detection sensitivity of targets in deep tissue. Here we characterize the bioluminescent properties of the newly synthesized luciferin analogue, AkaLumine-HCl. The bioluminescence produced by AkaLumine-HCl in reactions with native firefly luciferase is in the near-infrared wavelength ranges (λ_max_=677 nm), and yields significantly increased target-detection sensitivity from deep tissues with maximal signals attained at very low concentrations, as compared with D-luciferin and emerging synthetic luciferin CycLuc1. These characteristics offer a more sensitive and accurate method for non-invasive bioluminescence imaging with native firefly luciferase in various animal models.

## Abstract (web summary)

D-luciferin is the standard bioluminescent substrate for *in vitro* and *in vivo* imaging. Here the authors introduce AkaLumine-HCl, a soluble luciferin analogue with a near-infrared emission maximum, which allows deep tissue imaging at lower concentrations than D-luciferin.

## Graphical abstract

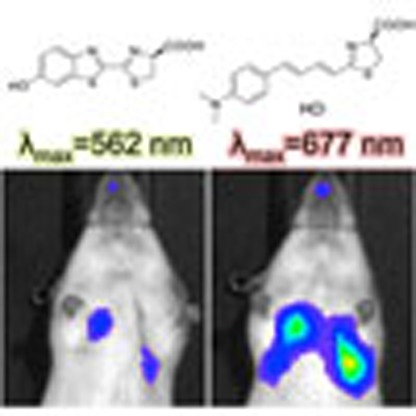

Bioluminescence is generated through an oxidation reaction between the enzyme luciferase and its substrate, luciferin^1^,^2^. In preclinical cancer research, bioluminescence imaging (BLI) with firefly luciferase (Fluc) and D-luciferin has become a standard to monitor tumour growth, protein–protein interactions and specific molecular activity in cell lines and mouse models^3^,^4^,^5^,^6^. The emission maximum (*λ*_max_) of bioluminescence produced by D-luciferin is 562 nm. In the visible light spectrum, haemoglobin (*λ*=∼415–577 nm) and melanin (*λ*<600 nm) dominate absorption, hindering the detection of signals emanating from deep tissues^7^,^8^. In recent times, cyclic alkylaminoluciferin (CycLuc1) (*λ*_max_=604 nm) demonstrated improved detection of targets *in vivo* by a significantly increased bioluminescence production over D-luciferin^9^. However, its short *λ*_max_ keep it from maximizing the detection sensitivity of targets in deep tissue.

To address this issue, efforts have been made to develop a BLI system that uses near-infrared (NIR) wavelength range of around 650–900 nm, where signal penetration through biological tissues is greatly improved^7^. NIR bioluminescence for *in vivo* BLI has been achieved with a synthetic D-luciferin analogue (*λ*_max_=706 nm)^10^ and bioluminescence resonance energy transfer in aminoluciferin-conjugated NIR fluorescence dyes (*λ*_max_<800 nm)^11^. However, these attempts have failed to show a significant improvement in the detection of targets *in vivo* when compared with D-luciferin, owing to their low bioluminescence production.

We previously synthesized a novel luciferin analogue, AkaLumine, by replacing the aromatic structure in D-luciferin with a benzothiazole moiety^12^. Notably, the *λ*_max_ of bioluminescence produced by AkaLumine is 675 nm, which displays high penetration *in vivo*. However, the high hydrophobicity of AkaLumine causes poor water solubility (<2 mM), which is a major drawback for its use *in vivo*. We thus screened water-soluble derivatives of AkaLumine. Here we report one of the derivatives, AkaLumine hydrochloride (AkaLumine-HCl) that is applicable for *in vivo* BLI. Akalumine-HCl emits light in the NIR wavelength ranges (*λ*_max_=677 nm) in reactions with native Fluc and yields increased detection sensitivity from deep-tissue targets with maximal signals attained at very low concentrations, as compared with D-luciferin and CycLuc1. These characteristics offer a more sensitive and accurate method for non-invasive BLI in various animal models using native Fluc.

## Results

### Characterization of AkaLumine-HCl

**Figure 1:**
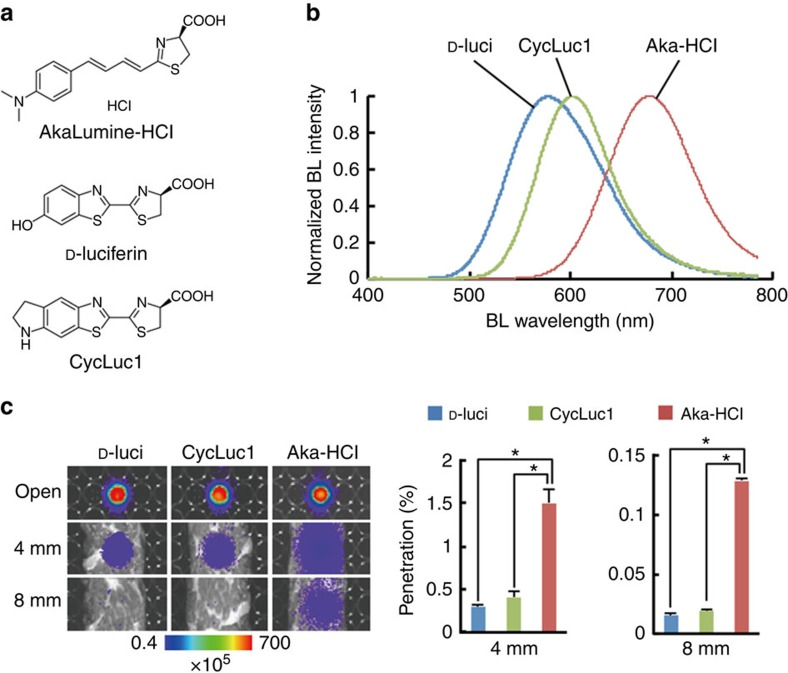
Properties of AkaLumine-HCl. (**a**) Chemical structures of AkaLumine-HCl, D-luciferin and CycLuc1. (**b**) Bioluminescence emission spectra of D-luciferin (D-luci), CycLuc1 or AkaLumine-HCl (Aka-HCl). (**c**) Biological tissue penetration efficiency of bioluminescence generated by D-luci, CycLuc1 or Aka-HCl. Penetration efficiency indicates the relative bioluminescence intensities of the wells covered with a biological tissue (4- or 8-mm thick) versus open wells (open). *n*=3, *P<0.05 (*t*-test). Error bars indicate s.e.m.

AkaLumine was able to detect targets in deep tissues of mouse tumour models with a significantly higher sensitivity than D-luciferin at the same concentration (Supplementary Fig. 1). To improve applicability of AkaLumine for *in vivo* imaging, we synthesized AkaLumine-HCl (Fig. 1a), which has high solubility in water (<40 mM). AkaLumine-HCl emitted NIR-shifted bioluminescence (*λ*_max_=677 nm) in reactions with Fluc (Fig. 1b) and greatly improved tissue penetration efficiency (Fig. 1c): AkaLumine-HCl bioluminescence showed 5- and 8.3-fold higher penetration than D-luciferin bioluminescence, and 3.7- and 6.7-fold higher penetration than CycLuc1 bioluminescence in 4- or 8-mm-thick tissue sections, respectively (Fig. 1c). A 60-fold higher concentration of D-luciferin was required to obtain signal intensity comparable to AkaLumine-HCl (150 μM versus 2.5 μM, respectively; Supplementary Fig. 2). These results suggest that AkaLumine-HCl is better suited for the detection of targets in deep tissue.

**Figure 2:**
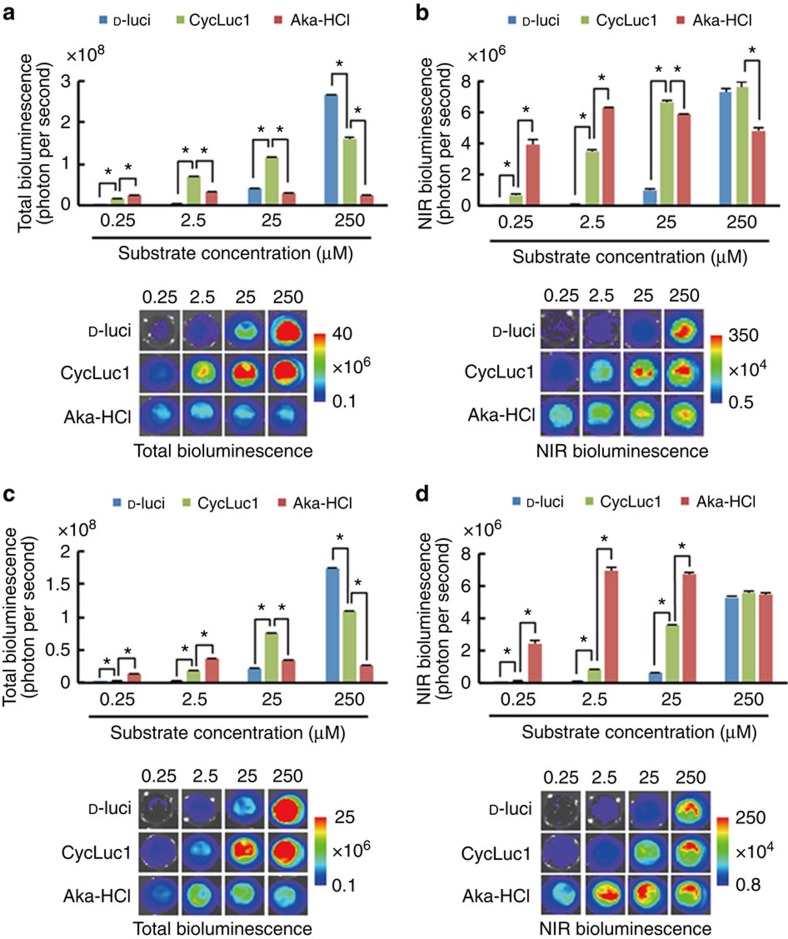
Cellular imaging using AkaLumine-HCl. Substrate dose dependency of total (**a**,**c**) or NIR (**b**,**d**) bioluminescence production in LLC/luc (**a**,**b**) or MDA-MB-231/luc (**c**,**d**) cells. The cells (4 × 10^5^ cells per well) were treated with the substrate at indicated concentrations. Images were acquired with 680±10 nm emission filter (right panels) or without a filter (left panels), to measure NIR bioluminescence or total bioluminescence signals, respectively. *n*=3, *P<0.05 (*t*-test). Error bars indicate s.e.m.

**Figure 3:**
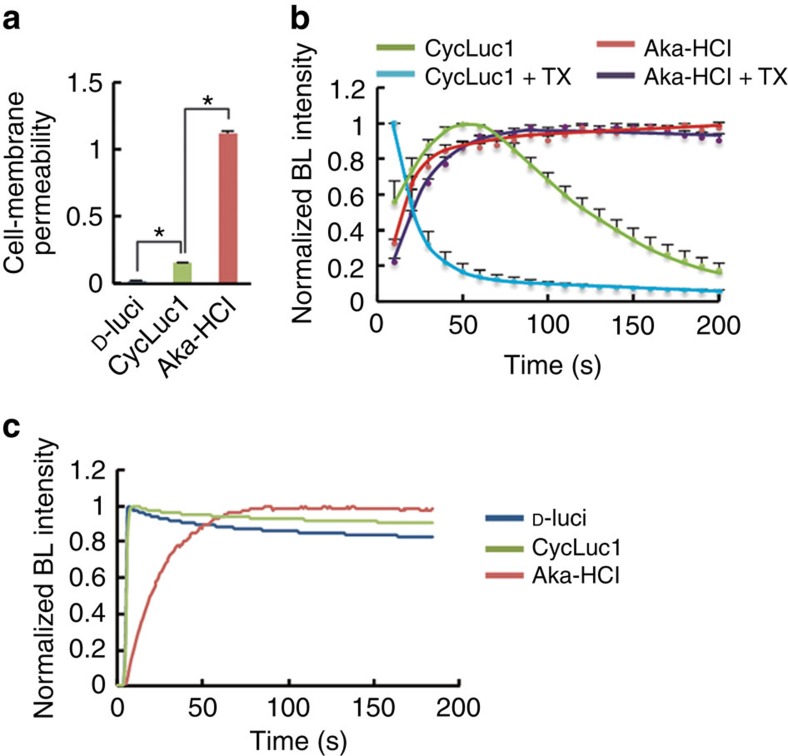
Cell-membrane permeability of AkaLumine-HCl. (**a**) Cell-membrane permeability of the substrates. Ratio of bioluminescence (BL) intensity indicates relative bioluminescence intensity of intact cells versus lysed cells treated with indicated substrates at 2.5 μM. *n*=3, *P<0.05 (*t*-test). (**b**) BL production in LLC/luc cells with or without pretreatment with 0.1% Triton X-100 (TX). BL intensity was measured every 10 s after addition of CycLuc1 or AkaLumine-HCl (Aka-HCl) (final concentration of 25 μM). BL intensity at each time point was normalized to the peak intensity. *n*=3. (**c**) BL production with recombinant Fluc protein. The substrates (final concentration of 25 μM) were reacted with recombinant Fluc proteins (20 μg ml^−1^). The reaction was initiated by the addition of ATP-Mg (final concentration of 80 μM). Data are representative of three independent experiments. Error bars indicate s.e.m.

To evaluate the properties of AkaLumine-HCl in BLI at the cellular level, bioluminescence signals from LLC/luc (mouse lung carcinoma) and MDA-MB-231/luc (human breast carcinoma) cancer cell lines with constitutive expression of Fluc were analysed after treatments with increasing concentrations of D-luciferin, CycLuc1 and AkaLumine-HCl (Fig. 2). Interestingly, in both LLC/luc and MDA-MB-231/luc cells treated with AkaLumine-HCl, the signal was maximal at lower concentrations (by 2.5 μM), whereas the bioluminescence generated by D-luciferin and CycLuc1 increased as concentrations increased further, not appearing to reach a maximal signal even at 250 μM (Fig. 2). The bioluminescent signals in the NIR range, which provide a better correlation with *in vivo* images, from AkaLumine-HCl were strong and less influenced by their concentrations, whereas the ones from CycLuc1 and D-luciferin showed a dose-dependent increase and were weak at lower concentrations that are commonly reached *in vivo* by ordinary mouse experiments (Fig. 2b,d). These results illustrate the differences in dose dependence among the three agents, with AkaLumine-HCl appearing to be optimally effective at much lower doses than the others, an effect particularly evident when imaging in the NIR range. Both AkaLumine-HCl and CycLuc1 showed less dose dependency than D-luciferin and decreased in bioluminescence production at a high concentration in the reaction with recombinant Fluc protein (Supplementary Fig. 3) probably due to their low *K*_m_ values (Table 1), which lead to saturation of Fluc with them at relatively low concentrations, and product inhibition effects, in which the mechanism is reported for D-luciferin^13^,^14^ but still unknown for CycLuc1 and AkaLumine-HCl. However, CycLuc1 showed a clear dose-dependent bioluminescence production with Fluc expressed in the cells (Fig. 2) at the same concentration range as the reaction with recombinant Fluc protein (Supplementary Fig. 3). This dose dependency of CycLuc1 is probably due to limited cell-membrane permeability, which is a major factor limiting enzyme–substrate interactions in cells, influencing bioluminescence production rate^15^,^16^,^17^. Comparison of bioluminescence production levels in intact and lysed cells shortly after treatments with the substrates revealed the differences in cell-membrane permeability among them: the cell-membrane permeability of AkaLumine-HCl was much greater than the others (Fig. 3a). Furthermore, pretreatment of the cells with Triton-X-100 greatly accelerated the bioluminescence production rate of CycLuc1 (Fig. 3b), revealing that the membrane permeability is the rate-limiting factor of the bioluminescence production by CycLuc1. On the other hand, AkaLumine-HCl showed similar bioluminescence production kinetics in intact or detergent-treated cells and in the reaction with recombinant Fluc protein (Fig. 3b,c). Taken together, small *K*_m_ value and high cell-membrane permeability may be key factors for distinct bioluminescence production by AkaLumine-HCl in cells. This property is a significant advantage over D-luciferin and CycLuc1 for bioluminescence animal imaging, as variation in substrate bioavailability among tissues is one of the main issues hindering the acquisition of quantitative information of targets located in different parts of the body. In addition, we also confirmed the linear correlation of signal output with increasing numbers of LLC/luc and MDA-MB-231/luc cells following treatment with 25 μM AkaLumine-HCl (Supplementary Fig. 4).

### Highly sensitive *in vivo* BLI using AkaLumine-HCl

**Figure 4:**
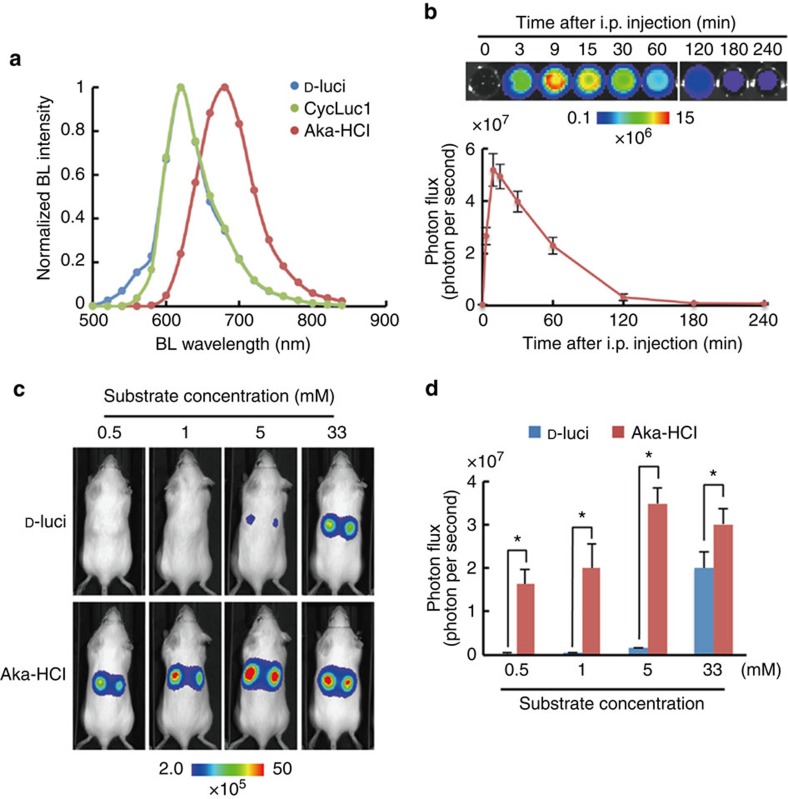
*In vivo* BLI of cancer cells using AkaLumine-HCl. (**a**) Bioluminescence spectrum from subcutaneous tumours. Bioluminescence (BL) intensity was measured with 18 filters (500–840 nm) in IVIS Spectrum after injection of D-luciferin (D-luci), CycLuc1 and AkaLumine-HCl (Aka-HCl) into mice bearing subcutaneous LLC/luc tumours. Date are representative of three independent experiments. (**b**) Half-life of AkaLumine-HCl in serum. Recombinant Fluc proteins were reacted with blood sampled at indicated time after intraperitoneal injection of 5 mM AkaLumine-HCl. Representative BL images (upper) and quantitative analysis (bottom) of BL intensity generated by reaction with recombinant Fluc proteins and AkaLumine-HCl in sampled blood are shown. *n*=3. (**c**) Representative BL images of LLC/luc subcutaneous tumours and (**d**) quantitative analysis of BL production 15 min after intraperitoneal injection of 100 μl of D-luciferin (D-luci) or AkaLumine-HCl (Aka-HCl) with indicated concentration. *n*=6, *P<0.05 (*t*-test). The substrates were injected to the same mouse in the order of D-luci and Aka-HCl at a 4-h interval. Error bars indicate s.e.m.

We then sought to assess the suitability of AkaLumine-HCl for *in vivo* BLI. AkaLumine-HCl emitted NIR-shifted bioluminescence from subcutaneous tumours with a similar spectrum to that obtained in the reactions with recombinant Fluc protein (Fig. 4a). In a time course of bioluminescence production *in vivo*, AkaLumine-HCl displayed a stable glow-type reaction at various injection doses and its bioluminescence production remained elevated longer at the higher concentrations (Supplementary Fig. 5); this temporally more stable bioluminescence production may be beneficial for acquiring images without influence of acquisition time. Furthermore, AkaLumine-HCl has a relatively longer half-life (∼40 min) in serum (Fig. 4b). These results suggest that AkaLumine-HCl would be a suitable substrate for *in vivo* imaging. To address this issue, we first compared the differences in *in vivo* detection sensitivity between bioluminescence signals generated by D-luciferin and AkaLumine-HCl in a subcutaneous tumour, which is one of the most easily detectable surface targets, after intraperitoneal injection with various doses of these substrates. AkaLumine-HCl was injected into the mice 4 h after D-luciferin injection, when D-luciferin bioluminescence was negligible (Supplementary Fig. 6a). The bioluminescence signals produced by AkaLumine-HCl were >40-fold higher than those of D-luciferin after injecting 1 mM substrates (Fig. 4c,d). This improvement in the detection of subcutaneous tumours is comparable to CycLuc1 (ref. 9). We then compared bioluminescence production between AkaLumine-HCl and CycLuc1 in subcutaneous tumours by intraperitoneal injection of 5 mM substrate into the same mice in order of CycLuc1 and AkaLumine-HCl at an 8- h interval, which was long enough to make CycLuc1 bioluminescence negligible (Supplementary Fig. 6b). Detection sensitivity of surface targets was comparable between 5 mM AkaLumine-HCl and CycLuc1 (Supplementary Fig. 7), reflecting the fact that the signals from the body surface are less influenced by tissue penetration efficiency and thus receive less benefit from NIR bioluminescence.

### Improved deep tissue imaging using AkaLumine-HCl

**Figure 5:**
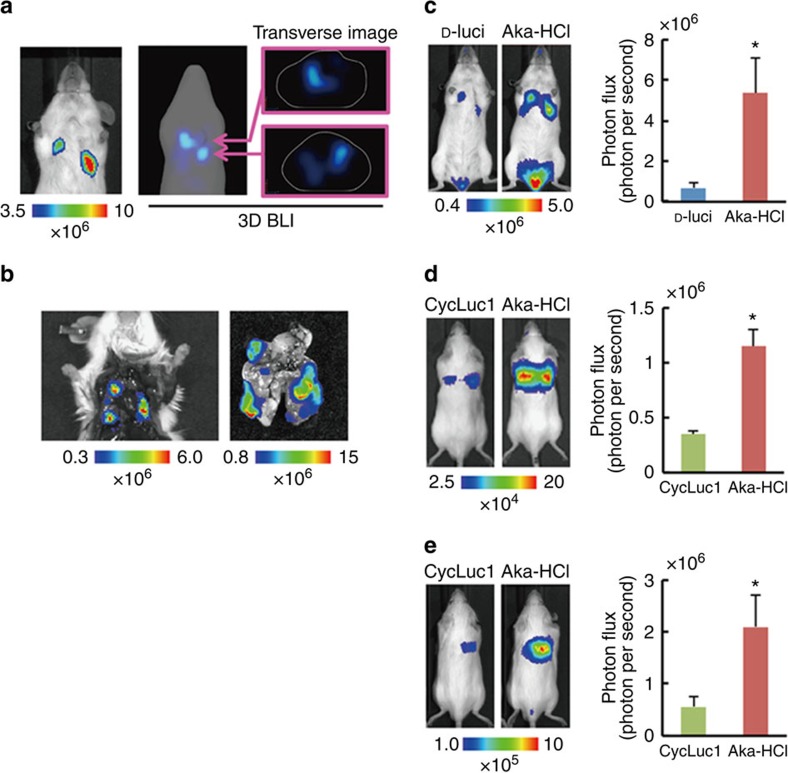
Highly sensitive *in vivo* BLI of deep tissue tumours. (**a**) Typical bioluminescence (BL) images of lung metastasis developed after intravenous injection of LLC/luc cells. Three-dimensional BLI (right panels) showed metastasis developed in deep lung tissues. The images were acquired after intraperitoneal injection of AkaLumine-HCl (33 mM). (**b**) *Ex vivo* BLI of metastatic lesions in the lung after removing sternum (left panel) and removed lung (right panel) of the mouse shown in **a**. (**c**) Representative BL images of LLC/luc lung metastasis (left panel) and quantitative analysis of BL production (right panel) at peak time after intraperitoneal injection of 100 μl of 33 mM substrates. *n*=7, *P<0.05 (*t*-test). (**d**) Representative BL images of LLC/luc disseminated in the lung (left panel) and quantitative analysis of BL production (right panel) at peak time after intraperitoneal injection of 100 μl of 5 mM substrates. *n*=8, *P<0.05 (*t*-test). Error bars indicate s.e.m. (**e**) Representative BL images of lung metastasis of LLC/luc (left panel) and quantitative analysis of BL production (right panel) at peak time after intraperitoneal injection of 100 μl of 5 mM substrates. *n*=8, *P<0.05 (*t*-test). Error bars indicate s.e.m.

We then address whether AkaLumine-HCl could improve the detection sensitivity for targets located in deep tissues such as metastasis in mouse lung, which is one of the most difficult tissues for optical imaging due to high scattering and absorption of light^18^, as well as its location deep within the body (Fig. 5a,b). Bioluminescence images were acquired from the same mice bearing LLC/luc lung metastases after intraperitoneal injection in order of 33 mM D-luciferin and AkaLumine-HCl at a 4-h interval. Notably, AkaLumine-HCl displayed greatly enhanced signals emanating from metastasis in the lung (Fig. 5c), as demonstrated by 8.1-fold higher with AkaLumine-HCl as compared with D-luciferin administration (Fig. 5c). To evaluate the advantage of AkaLumine-HCl over CycLuc1 in detecting targets in deep tissue, we then compared bioluminescence images between AkaLumine-HCl- and CycLuc1-treated mice 15 min after intravenous injection of LLC/luc cells at the dose of 5 mM, which is the maximum concentration of CycLuc1 due to its low water solubility (Fig. 5d). AkaLumine-HCl exhibited 3.3-fold increase in detection sensitivity of disseminated cancer cells in the lung compared with CycLuc1 (Fig. 5d). The advantage of AkaLumine-HCl was further confirmed by imaging the same mice bearing lung metastasis after intraperitoneal injection of 5 mM substrates in order of CycLuc1 and AkaLumine-HCl (Fig. 5e), and in the inverse order (Supplementary Fig. 8) at an 8-h interval. AkaLumine-HCl displayed about fourfold increase in signals from lung metastasis compared with CycLuc1 (Fig. 5e and Supplementary Fig. 8).

## Discussion

In this study, we demonstrate that the bioluminescence produced by AkaLumine-HCl achieved considerable penetration depths (Figs 1c and 5c–e) and exhibited small temporal fluctuation (Supplementary Fig. 5), much less influenced by its concentration (Figs 2 and 4d). The properties that enable more sensitive detection of targets in deep tissues and more accurate quantitative comparison of targets localizing to different parts of the body are significant advantages of AkaLumine-HCl over D-luciferin and CycLuc1, and therefore AkaLumine-HCl might become the preferred BLI substrate in preclinical studies. The small temporal fluctuation in signal production by AkaLumine-HCl in a mouse model (Supplementary Fig. 5) may be achieved by its properties of lower saturation concentration (Fig. 2), high membrane permeability (Fig. 3) and relatively longer circulation half-time in the serum (Fig. 4b). These properties may contribute to having the reaction rate remain constant in tumour cells for relatively long periods of time, leading to more accurate and efficient quantitative analysis of targets from many mice in limited time.

In this study, we demonstrated that AkaLumine-HCl significantly improved target detection sensitivity in deep lung metastases (Fig. 5c–e) by overcoming the fundamental limitation of short emission wavelength from D-luciferin and CycLuc1. Of note, a recent study detailed the synthesis of infra-luciferin (iLH_2_), which emits NIR bioluminescence of the longest-known wavelength via its reaction with mutant Fluc^10^. However, the iLH_2_ signal output was exceedingly low when compared with that of D-luciferin and, therefore, iLH_2_ requires chemical optimization for practical use in biological studies. AkaLumine-HCl exhibits a considerably high NIR bioluminescence production with native Fluc that is currently used in general *in vivo* BLI applications. As such, AkaLumine-HCl would be immediately available for use in a broad range of biological studies that use BLI in small animal models.

## Methods

### Synthesis of AkaLumine-HCl

AkaLumine was synthesized as previously described^12^. To prepare AkaLumine-HCl, 4 M HCl in Dioxane (0.5 ml) was added to a suspending solution of the AkaLumine (20 mg, 0.066 mmol). This mixture solution was centrifuged after vortex for 10 min. The supernatant and the precipitate were then subjected to solid–liquid separation. The obtained precipitate was washed with 1 ml ethylacetate three times and dried in vacuum to obtain the AkaLumine-HCl (20 mg, 0.059 mmol) as a light purple solid; 85% ee from chiral HPLC (retention time of L-isomer: 16.8 min, D-isomer: 17.6 min); ^1^H NMR (500 MHz, DMSO-*d*_6_):*d* 7.67 (*dd*, *J*=14.9, 11.5 Hz, 1H), 7.59 (*d*, *J*=9.16 Hz, 2H), 7.38 (*d*, *J*=15.1 Hz, 1H), 7.19 (*dd*, *J*=14.9, 11.5 Hz, 1H), 6.92 (br, 2H), 6.81 (*d*, *J*=14.9 Hz, 1H), 5.47 (*dd*, *J*=10.3, 5.5 Hz, 1H), 3.98 (*dd*, *J*=11.5, 5.5 Hz, 1H), 3.87 (*dd*, *J*=11.5, 10.3 Hz, 1H), 3.04 (*s*, 6H); ^13^C NMR (125 MHz, DMSO-*d*_6_) *d* 182.8, 169.8, 154.3, 150.9, 148.6, 131.0, 126.1, 123.6, 114.5, 66.5, 41.5, 34.39.

The optical purity of synthesized luciferin analogues was analysed by Thermo LC-MS (LCQ Fleet) using a chiral column (Daicel Chemical Industries, OZ-RH, 5 mm, 4.6–150 mm) with a linear gradient of 10–90% acetonitrile in 0.05% TFA in H_2_O over 30 min (flow rate 0.5 ml min^−1^) as eluent. ^1^H and ^13^C NMR spectra were recorded on JEOL ECA 500 instruments. Chemical shifts are reported in parts per million (*d*) downfield from internal tetramethylsilane (*d* ¼0) and coupling constants in Hertz. Infrared spectra were measured by Nicolet 6700 FT-IR Spectrometer using attenuated total reflection methods. AkaLumine-HCl (trade name: TokeOni, 808350-5MG) can be obtained from Sigma-Aldrich (St Louis, MO, USA).

### Measurement of bioluminescence emission spectra

Bioluminescence emission spectra of D-luciferin (Promega, Madison, WI, USA), CycLuc1 (AOBIOUS INC., Gloucester, MA, USA) and AkaLumine-HCl were measured using an ATTO AB-1850 spectrophotometer (ATTO Co. Ltd, Tokyo, Japan). A reaction mixture was prepared by mixing 5 μl of a substrate (100 μM), 5 μl of QuantiLum Recombinant Luciferase solution (1 mg ml^−1^) (Promega) and 5 μl of potassium phosphate buffer (500 mM, pH 8.0). Luminescence reactions were then initiated by injecting 10 μl of ATP-Mg (200 μM) into the reaction mixture. Bioluminescence emission spectra were measured in 1 nm increments from 400 to 780 nm using 3 min of integration time.

### Measurement of *K*_m_ value

The bioluminescence intensities of D-luciferin, CycLuc1 and AkaLumine-HCl were measured in same conditions with measurement of bioluminescence spectrum, except substrate concentration. Final concentrations of the substrates were varied from 0.2 to 500 μM and 0.1 to 100 μM, respectively. *K*_m_ and *V*_max_ values of the substrates were determined from the integrated value of the bioluminescence intensity and calculated by the Lineweaver–Burk plots by using the Enzyme Kinetics Wizard in the commercially available SigmaPlot 9.0 software package (Systat Software Inc., San Jose, CA, USA).

### Bioluminescence transmission assay using biological tissues

Bioluminescence signal from wells was measured with IVIS Spectrum (PerkinElmer, Boston, MA, USA) 1 min after the substrate (final concentration of 2.5 μM for D-lucfierin and AkaLumine-HCl, 50 nM for CycLuc1) was reacted with recombinant Fluc proteins (20 μg ml^−1^) in the presence of ATP-Mg (final concentration of 20 μM) in a black 96-well plate. A biological tissue (4-mm-thick sliced beef) was placed on the wells, to measure bioluminescence signal through the tissue, followed by acquiring bioluminescence images through two layers of the biological tissue. The following conditions were used for image acquisition: exposure time=10 s, binning=medium: 8, field of view=12.9 × 12.9 cm, and *f*/stop=1. The bioluminescence images were analysed by Living Image 4.3 software (PerkinElmer) specialized for IVIS system. Penetration efficiency (%) was calculated by dividing the signal intensities of the beef-covered wells by those of the corresponding uncovered wells.

### Isolation of cancer cell lines stably expressing luc reporters

The murine lung carcinoma cell LLC, human breast cancer cell MDA-MB-231 and human prostate cancer cell PC-3 were obtained from ATCC (Rockville, MD, USA). LLC/luc and MDA-MB-231/luc cells were isolated after transfection with plasmid pEF/luc by calcium phosphate method as previously described^19^,^20^. PC-3/κB-luc was also isolated as previously described^21^. The cells were maintained at 37 °C in 5% FCS-DMEM (Nacalai Tesque, Kyoto, Japan) supplemented with penicillin (100 units per ml) and streptomycin (100 μg ml^−1^) and regularly checked for mycoplasma contamination by a mycoplasma check kit (Lonza, Basel, Switzerland). All the cell lines were independently stored and recovered from the original stock every time for each experiment.

### *In vitro* BLI

The substrates were reacted with LLC/luc or MDA-MB-231/luc cells (4 × 10^5^ cells per well) in a black 96-well plate. Bioluminescence was measured using IVIS Spectrum 1 min after adding the substrates. The following conditions were used for image acquisition: open for total bioluminescence or 680±10 nm of an emission filter for NIR bioluminescence, exposure time=10 s, binning=medium: 8, field of view=12.9 × 12.9 cm and *f*/stop=1. The bioluminescence images were analysed by Living Image 4.3 software (PerkinElmer) specialized for IVIS system.

### Cell-membrane permeability assay

Bioluminescence from lysed or intact LLC/luc (10^5^ cells) was measured in a black 96-well plate using IVIS Spectrum. Lysed cells were prepared with 2 × Passive Lysis Buffer (Promega) supplemented with Tris-HCl (20 mM), EDTA (0.2 mM), ATP-Mg (500 nM), BSA (6 mg ml^−1^) and dithiothreitol (33 mM). The measurement was performed 1 min after adding the substrates (final concentration of 2.5 μM). The following conditions were used for image acquisition: open emission filter, exposure time=10 s, binning=medium: 8, field of view=12.9 × 12.9 cm and *f*/stop=1. The bioluminescence images were analysed by Living Image 4.3 software (PerkinElmer) specialized for IVIS system. Bioluminescence emission from LLC/luc (5 × 10^5^ cells) with or without pretreatment of 0.1% Triton-X-100 for 3 min was measured every 10 s for 200 s using a luminometer GL-210A (Microtec Co., Ltd, Chiba, Japan). Bioluminescence measurement was started by injection of the substrate (final concentration of 25 μM).

### Mice

C57B/6 albino mice (female and male) or severe combined immunodeficient mice (male) were obtained from Charles River Laboratory Japan (Yokohama, Japan). All mice used were littermates or age-matched (7 weeks of age) females or males, were provided access to food and water *ad libitum* and were housed in the animal facilities at Tokyo Institute of Technology.

All the experimental procedures using mice were approved by the Animal Experiment Committees of Tokyo Institute of Technology (authorization numbers 2010006-3 and 2014005) and carried out in accordance with relevant national and international guidelines.

### Tumour models

For subcutaneous tumour models, LLC/luc (3 × 10^5^ cells per 10 μl) or PC-3/κB-luc (1 × 10^6^ cells per 10 μl) suspended in PBS was mixed with an equal volume of Geltrex (Invitrogen) and subcutaneously or intratibially injected into C57B/6 albino mice (female) or severe combined immunodeficient mice (male), respectively. The experiments were performed 4 days after engraftment. For a model with disseminated cancer cells in the lung, C57B/6 albino mice (male) were intravenously injected with LLC/luc (1 × 10^5^ cells per 100 μl PBS) 15 min before *in vivo* BLI. For lung metastasis model, LLC/luc (5 × 10^5^ cells per 100 μl) suspended in PBS was injected from tail vein of C57B/6 albino mice (male). The experiments were performed 10–20 days after intravenous injection. These tumour models are well established and tumour growth is stable. Therefore, six samples are adequate sample size for evaluation of tumour growth in each experiment.

### *In vivo* BLI

Bioluminescence images of subcutaneous tumours were acquired with IVIS Spectrum 15 min (unless otherwise indicated) after intraperitoneal injection with indicated amount of the substrates. As bioluminescence intensities from lung metastasis peaked at various time after a substrate injection, bioluminescence images of lung metastasis were sequentially acquired with IVIS Spectrum every 3 min for 30 min after intraperitoneal injection with the substrates and the highest bioluminescence intensities among the acquired images were selected for analysis. For comparing bioluminescence production between different substrates using the same mice, the images for AkaLumine-HCl were acquired 4 and 8 h after injection of D-luciferin and CycLucl, respectively. The following conditions were used for image acquisition: open emission filter, exposure time=60 s, binning=medium: 8, field of view=12.9 × 12.9 cm and *f*/stop=1. For three-dimensional BLI in lung metastasis model, a mouse injected with AkaLumine-HCl was subjected to BLI with three different wavelengths (660±10, 680±10 and 700±10 nm) of bioluminescence filters. The following conditions were used for imaging acquisition: exposure time=60 s, binning=medium: 8, field of view=12.9 × 12.9 cm and *f*/stop=1. The bioluminescence images were analysed by Living Image 4.3 software (PerkinElmer) specialized for IVIS system.

### *Ex vivo* BLI

A mouse was scarified immediately after *in vivo* BLI by using AkaLumine-HCl and the lung was removed. Bioluminescence image of the lung was obtained with the following conditions: open emission filter, exposure time=30 s, binning=medium: 8, field of view=12.9 × 12.9 cm and *f*/stop=1. The bioluminescence images were analysed by Living Image 4.3 software (PerkinElmer) specialized for IVIS system.

### Measurement of AkaLumine-HCl half-life in serum

Five microlitres of blood was sampled from tail vein and then mixed with 45 μl PBS, 25 μl recombinant Fluc protein (20 μg ml^−1^) and 25 μl ATP-Mg (80 μM) in a black 96-well plate, to measure bioluminescence intensity by IVIS Spectrum. The following conditions were used for image acquisition: exposure time=10 s, binning=medium, field of view=12.9 × 12.9 and *f*/stop=1. The bioluminescence images were analysed by Living Image 4.3 software.

### Statistical analysis

Data are presented as means±s.e.m. and were statistically analysed with a two-sided Student's *t*-test. *P*-values<0.05 were considered statistically significant.

### Data availability

The data that support the findings of this study are available from the corresponding author on request.

## Additional information

**How to cite this article**: Kuchimaru, T. *et al.* A luciferin analogue generating near-infrared bioluminescence achieves highly sensitive deep-tissue imaging. *Nat. Commun.* 7:11856 doi: 10.1038/ncomms11856 (2016).

## Supplementary Material

Supplementary Information Supplementary Figures 1-8

## Figures and Tables

**Table 1:** Parameters of enzymatic reaction of luciferase substrate.

	***K***_**m**_**±s.e.m. (μM)**	***V***_**max**_**±s.e.m. (× 10**^**6**^**)**
D-luci	107±14.7	13.8±0.71
CycLuc1	1.06±0.06	7.00±0.09
Aka-HCl	2.06±0.63	2.07±0.23

Aka-HCl, AkaLumine-HCl, CycLuc1, cyclic alkylaminoluciferin; D-luci, D-luciferin.

*K*_m_ and *V*_max_ values of D-luci, CycLuc1 and Aka-HCl. These values were calculated from reactions with recombinant Fluc protein and the substrate in the presence of Mg-ATP.
