# Supplementary material for: A luciferin analogue generating near-infrared bioluminescence achieves highly sensitive deep-tissue imaging
Source: Nat Commun. 2016 Jun 14;7:11856. doi: 10.1038/ncomms11856 (PMC4911627; doi:10.1038/ncomms11856)
Supplement: Supplementary Information — Supplementary Figures 1-8 [file ncomms11856-s1.pdf]

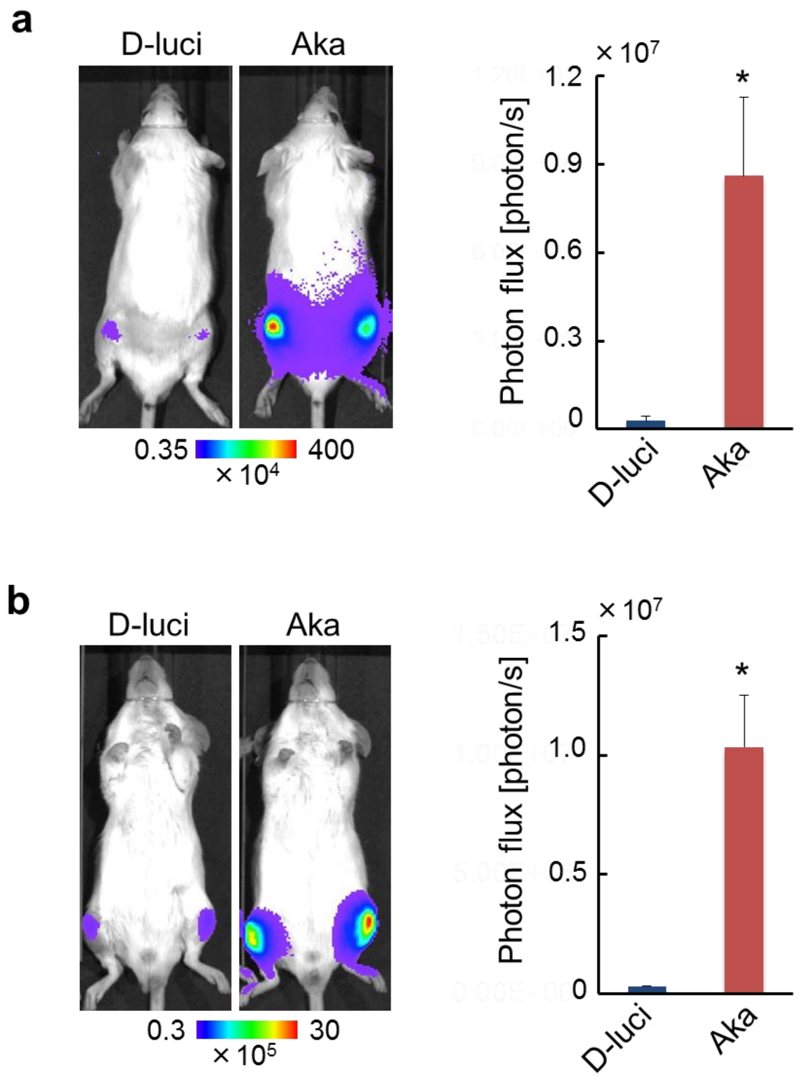

Supplementary Figure 1 Increased detection sensitivity of luciferase *in vivo* by AkaLumine. Representative bioluminescence images (left panels) of PC-3/κB-luc in (**a**) subcutaneous xenograft and (**b**) bone metastasis models and quantitative analysis of bioluminescence production (right panels) 15 min after intraperitoneal injection of 100 μL of 500 μM D-luciferin (D-luci) or AkaLumine (Aka). n=6, *p<0.05 (t-test). Error bars indicate s.e.m.

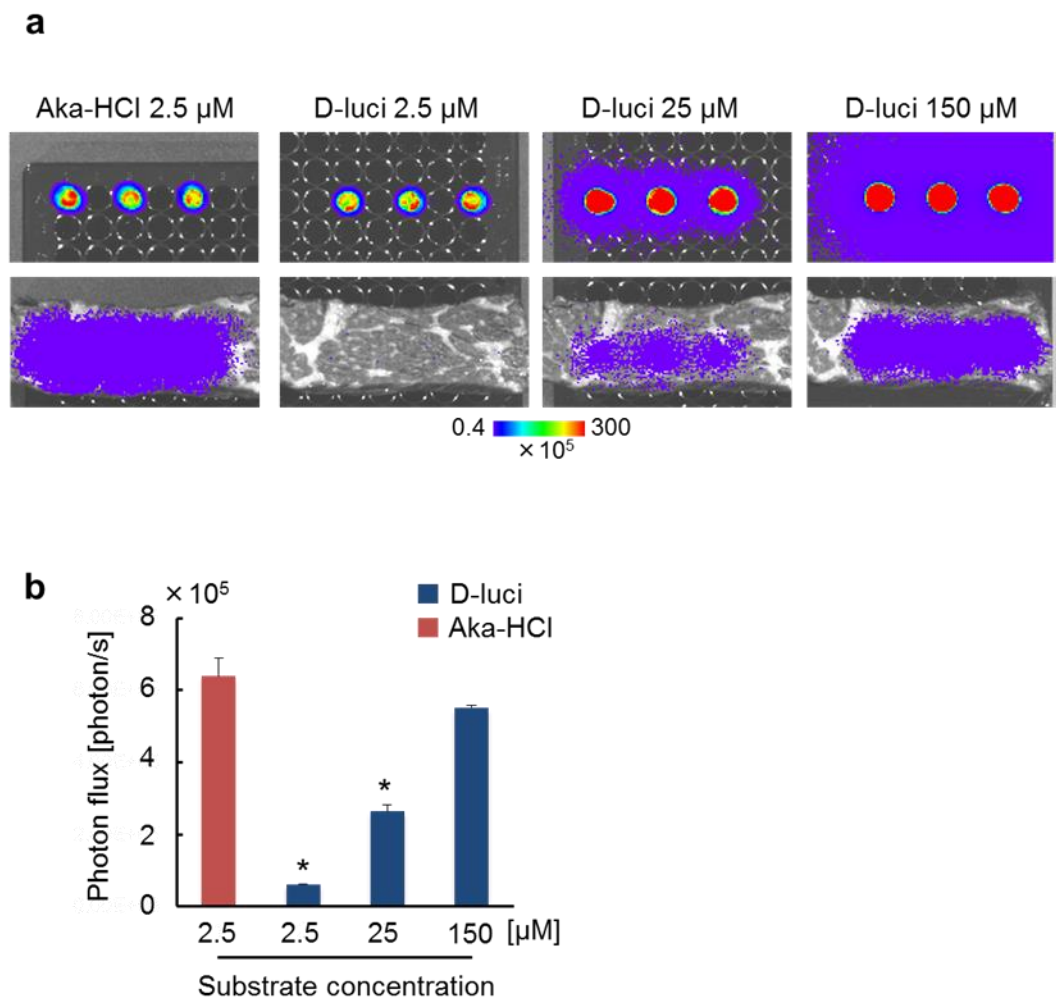

Supplementary Figure 2 Biological tissue penetration efficiency of bioluminescence. (**a**) Bioluminescence images of recombinant Fluc protein in a 96-well plate covered without (top panels) or with (bottom panels) biological tissues (8-mm thick). Recombinant Fluc protein (20 μg per mL) was mixed with indicated substrates in the presence of 20 μM ATP-Mg. (**b**) Quantitative analysis of transmitted light of the bottom images of (a). n=3, *$p<0.05$ (t-test) to 2.5 μM Aka-HCl. Error bars indicate s.e.m.

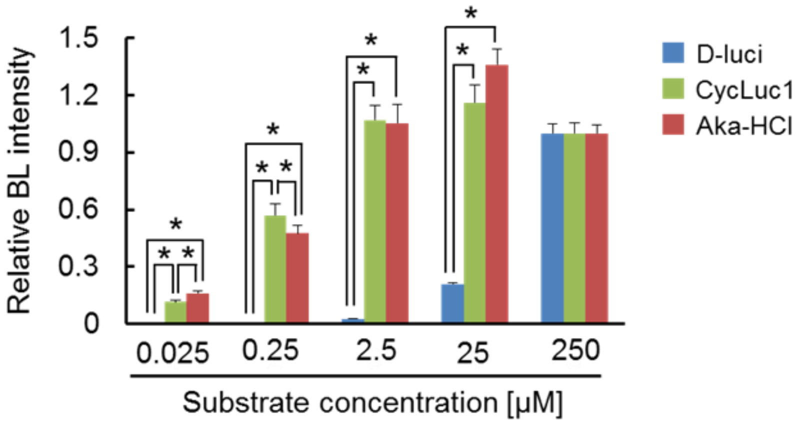

Supplementary Figure 3 Substrate dose-dependency of bioluminescence production with recombinant Fluc proteins. Recombinant Fluc protein (3 μg per mL) was mixed with indicated substrates in the presence of 80 μM ATP-Mg in a 96 well-plate. Bioluminescence (BL) intensity was measured using IVIS-Spectrum with a open filter. Relative BL intensity was calculated by dividing BL intensity of indicated concentration with that of 250 μM. n=3, *p<0.05 (t-test). Error bars indicate s.e.m.

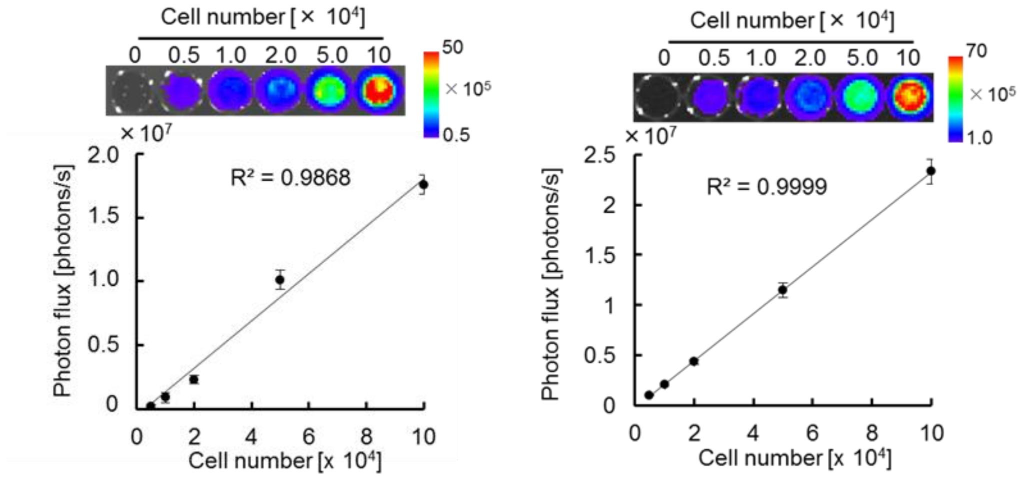

Supplementary Figure 4 Cellular imaging with AkaLumine-HCl. Cell number-dependent bioluminescence intensity of LLC/luc (left panel) and MDA-MB-231/luc cells (right panel). Indicated number of the cells were treated with 25 μM AkaLumine-HCl. n=3. Error bars indicate s.e.m.

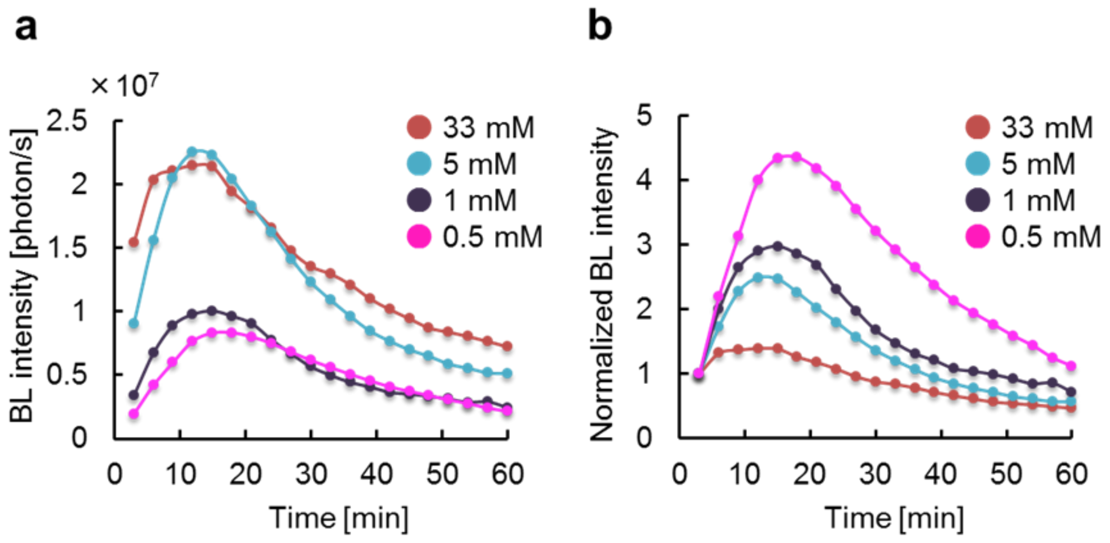

Supplementary Figure 5 Time course of bioluminescence production *in vivo*. Representative time course of bioluminescence (BL) production from subcutaneous tumors of LLC/luc. (**a**) BL intensity were measured every 3 min after i.p. injection of 100 μL AkaLumine-HCl at indicated concentration. (**b**) BL intensity at each time point was normalized to the ones obtained at 3 min after substrate injection. Data are representative of three independent experiments.

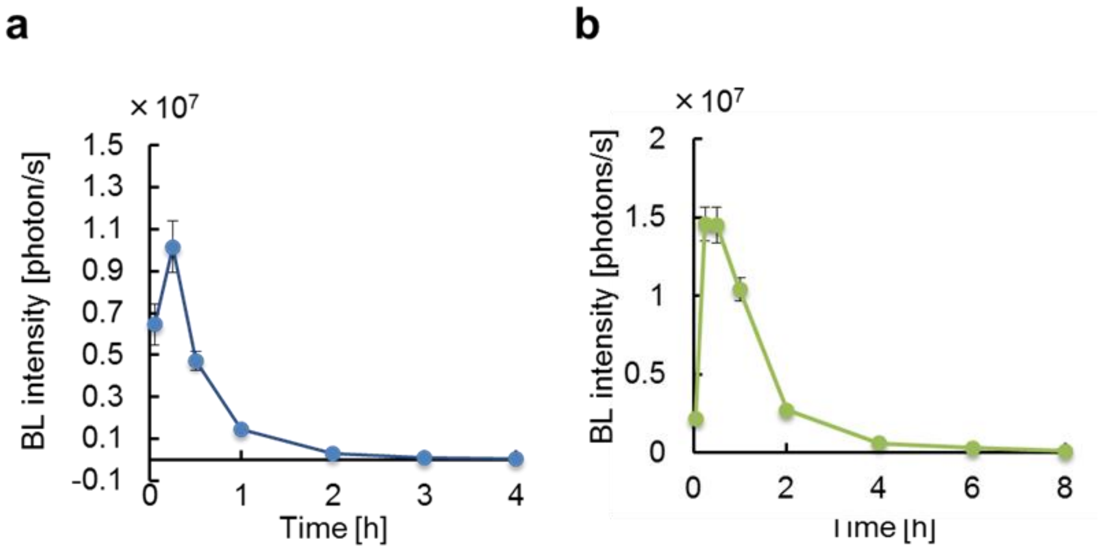

Supplementary Figure 6 Clearance of bioluminescence from subcutaneous tumor tissue. (**a**) Bioluminescence intensity was measured at 3, 15, 30, 60, 120, 180 and 240 min after intraperitoneal injection with 33 mM D-luciferin. (**b**) Bioluminescence intensity was measured at 3, 15, 30, 60, 240, 360 and 480 min after intraperitoneal injection with 5 mM CycLuc1 into mice with subcutaneous tumors of LLC/luc. n=4.

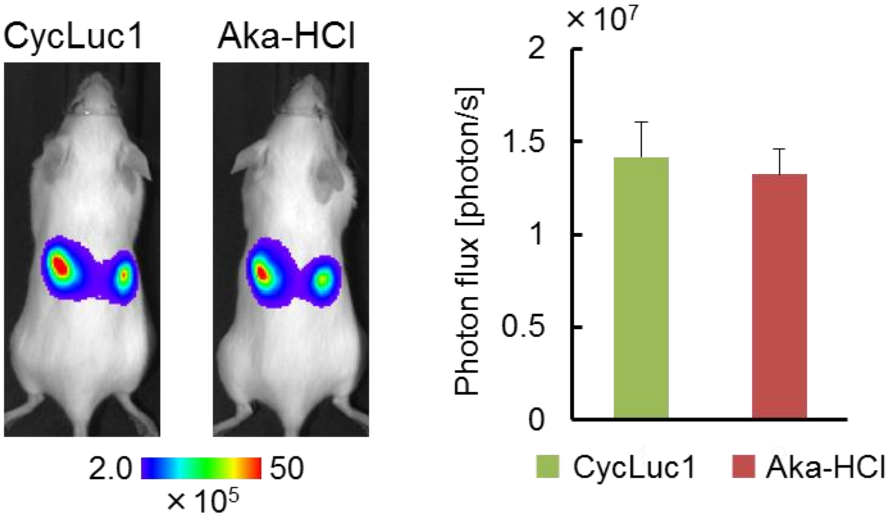

Supplementary Figure 7 Bioluminescence production in subcutaneous tumors by CycLuc1 and AkaLumine-HCl. Representative bioluminescence images (left panel) and quantitative analysis (right panel) of bioluminescence imaging of LLC/luc subcutaneous tumors after i.p. injection of the 5 mM substrates into the same mice in order of CycLuc1 and AkaLumine-HCl (Aka-HCl) with 8-h interval. n=6.

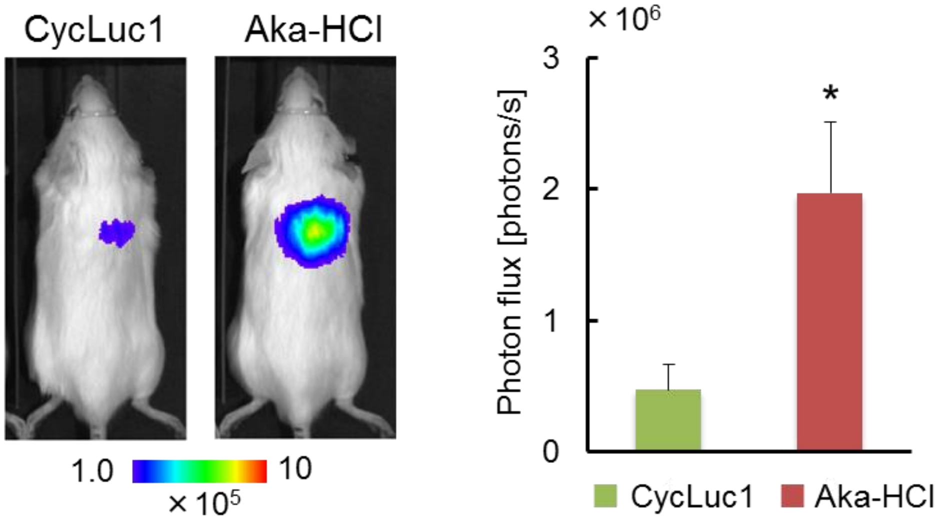

Supplementary Figure 8 Bioluminescence imaging of lung metastasis. Representative images (left panel) and quantitative analysis (right panel) of bioluminescence imaging of lung metastasis after i.p. injection of the 5 mM substrate in reverse order of Fig. 5e. n=8, *p<0.05.
